# Physiological Functions of Threonine in Animals: Beyond Nutrition Metabolism

**DOI:** 10.3390/nu13082592

**Published:** 2021-07-28

**Authors:** Qi Tang, Peng Tan, Ning Ma, Xi Ma

**Affiliations:** State Key Laboratory of Animal Nutrition, College of Animal Science and Technology, China Agricultural University, Beijing 100193, China; tangqi610@163.com (Q.T.); tanpeng1995@cau.edu.cn (P.T.); maningrebecca@cau.edu.cn (N.M.)

**Keywords:** threonine, metabolism, physiological effects, nutrition, intestinal health

## Abstract

Threonine (Thr), an essential amino acid for animals and the limiting amino acid in swine and poultry diets, which plays a vital role in the modulation of nutritional metabolism, macromolecular biosynthesis, and gut homeostasis. Current evidence supports that the supplementation of Thr leads to benefits in terms of energy metabolism. Threonine is not only an important component of gastrointestinal mucin, but also acts as a nutritional modulator that influences the intestinal immune system via complex signaling networks, particularly mitogen-activated protein kinase (MAPK) and the target of the rapamycin (TOR) signal pathway. Threonine is also recognized as an indispensable nutrient for cell growth and proliferation. Hence, optimization of Thr requirement may exert a favorable impact on the factors linked to health and diseases in animals. This review focuses on the latest reports of Thr in metabolic pathways and nutritional regulation, as well as the relationship between Thr and relevant physiological functions.

## 1. Introduction

Threonine (Thr), otherwise known as α-amino-β-hydroxybutyric acid, is an indispensable amino acid in animals that must be obtained from the diet [1]. It is also identified as the second limiting amino acid for finishing pigs fed a corn-soybean meal-based diet, and the third limiting amino acid in corn-soybean meal-based diets for broilers [2,3]. Threonine is an important bioactive molecule that has vital mediation effects on protein synthesis, energy metabolism, and nutrient absorption [2,4]. Many prior studies have focused on the requirements, physiological function, and metabolism pathway of Thr [5]. It has been reported that appropriate dietary Thr levels can promote animal growth, enhance immune function, and maintain intestinal health [6,7]. Threonine is an efficient nutritional modulator affecting nutrition metabolism. Threonine supplementation has been shown to enhance hepatic lipid metabolism, and it has been previously reported that Thr deficiency may induce hepatic triglyceride accumulation [8]. Threonine exerts a protective effect on lipid metabolic disorders by regulating the lipogenesis signaling pathway and the expression of thermogenic genes [9]. More importantly, a limited number of studies have indicated that Thr plays an indispensable role in physiological regulation in many processes besides simple nutrition, particularly in cell growth and proliferation, including in embryonic stem cells (ESCs) [10,11]. Embryonic stem cells are a kind of pluripotent stem cell, which can be induced to differentiate into multiple cell types. Embryonic stem cells have the potential to treat various diseases and injuries in animals, such as maintaining intestinal homeostasis and repairing liver injury [12,13]. Recent findings demonstrated that Thr is an effective metabolic fuel for mouse ESC self-renewal, as it provided a carbon source for macromolecular biosynthesis and epigenetic regulation [14].

Recently, a relationship between dietary Thr and animal intestinal health and function has been reported. An isotope tracer study showed that about 40–60% of dietary Thr is extracted by the gut during the first pass metabolism, which is mostly used for the synthesis of mucosal proteins, accounting for 71% of total Thr usage [15]. Threonine maintains intestinal homeostasis by acting on intestinal morphology, microorganisms, barrier, and immune function. When the intestinal tract is in an inflammatory state, Thr is used to regulate immune cell differentiation, cytokine expression, and immune-related signaling cascades, e.g., mitogen-activated protein kinase (MAPK), the target of rapamycin (TOR), to maintain intestinal health [16,17]. The scope of this review is to illustrate the Thr metabolism, influences, and underlying mechanism of Thr supplementation on the various physiological functions in animals.

## 2. The Metabolic Pathway of Thr

**Figure 1:**
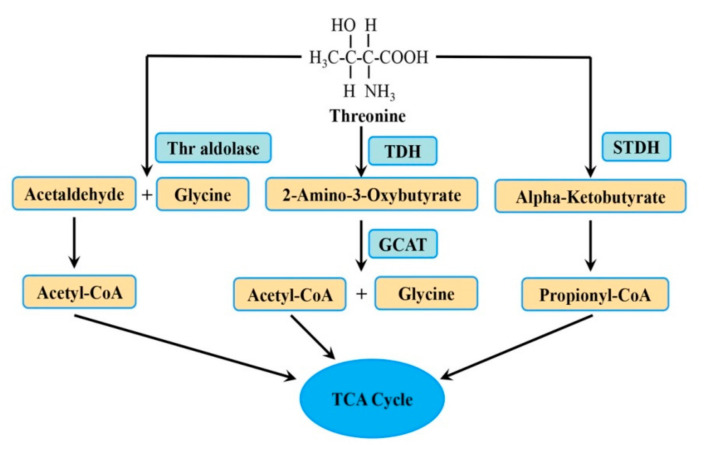
The metabolic pathway of Thr. Thr is metabolized to different intermediates and end products by three enzymes. Abbreviations: GCAT: 2-amino-3-oxobutyrate CoA ligase; STDH: threonine dehydratase; TCA: tricarboxylic acid cycle; TDH: threonine dehydrogenase; Thr: threonine.

Threonine mainly serves as a substrate for protein synthesis, particularly mucin. In addition, Thr can enter the catabolic pathway, where it can be metabolized to a variety of important products (glycine, acetyl CoA, pyruvate) that play a crucial role in host metabolism. Threonine undergoes three different metabolic pathways (Figure 1). The catabolism of Thr follows the glycine-independent or glycine-dependent pathway. One study found that under fasting and starvation conditions, Thr is catalyzed to α-ketobutyric acid and ammonia by liver threonine dehydratase (STDH) via the glycine-independent pathway, after which the ketobutyrate is decarboxylated to form propionyl-CoA [18]. Threonine dehydrogenase (TDH) and STDH are highly expressed in the pancreas and liver, respectively. However, threonine aldolase has a low level of enzymatic activity in the liver, and is highly expressed in the prostate [19,20]. TDH and threonine aldolase are in the glycine-dependent pathway. TDH is a key enzyme in Thr metabolism. It converts Thr to 2-amino-3-oxybutyrate, an unstable intermediate that is then degraded to acetyl-CoA and glycine by 2-amino-3-oxobutyrate CoA ligase (GCAT). Subsequently, acetyl-CoA enters the tricarboxylic acid (TCA) cycle, where it helps to produce energy. Moreover, threonine aldolase metabolizes Thr to glycine and acetaldehyde [1]. It is worth noting that the pathway of Thr metabolism may be different depending on the physiologic state. In infants, Thr is exclusively degraded by STDH [21], but in addition to the STDH pathway (glycine-independent), 7–10% of total Thr is catabolized by the TDH pathway in adults. This difference is due to the higher glycine requirements in infants compared to adults [22].

## 3. Role of Thr in Nutrition Metabolism

### 3.1. Lipid Metabolism

Essential amino acids have a close connection with the lipid metabolism, whereby certain amino acids (methionine, leucine, and isoleucine) can affect lipid deposition [23]. Thr similarly takes part in regulating lipid metabolism and deposition [24]. Thr supplementation has been shown to enhance hepatic lipid metabolism, while Thr deficiency may induce hepatic triglycerides accumulation, as shown in previous research [8]. Research on Pekin ducks found that dietary Thr shortage increased the expression of genes encoding 3-oxoacyl-ACP synthase (*OXSM*), very-long-chain fatty acids protein 7 (*ELOVL7*), and long-chain fatty acyl-CoA ligase (*ACSBG2*), which were related to fatty acid and triglyceride synthesis. However, genes participating in fatty acid oxidation, such as acyl-CoA dehydrogenase, acyl-CoA dehydrogenase family member 11 (*ACAD11*) and cytochrome P450 (*CYP4B*), as well as genes associated with fatty acid and triglyceride transport, acyl-CoA binding protein (*DBI*), apolipoprotein D (*APOD*), and microsomal triglyceride transfer protein (*MTTP*), were downregulated under Thr deficiency in Pekin ducks [24]. The equilibrium between lipogenesis and lipolysis determines lipid homeostasis [25]. Lipogenesis is the process by which preadipocytes develop into mature adipocytes, which is modulated by adipogenic transcription factors including peroxisome proliferator-activated receptors (PPARs) [26]. PPARγ plays a critical role in adipocyte differentiation, lipid metabolism, and insulin sensitivity, and regulates gene networks associated with glucose homeostasis, such as by boosting the expression of glucose transporter type 4 (Glut4) and c-Cbl–associated protein (CAP) [27]. Threonine supplementation in a high-fat diet may decrease lipid deposition by regulating the PPARγ signaling pathway [9]. Uncoupling protein-1 (*UCP1*) is a thermogenetic-related gene expressed in brown adipose tissue, which can disperse energy as heat to fight obesity [28]. *UCP1* expression was decreased in the brown adipose tissue of obese mice, but this was restored by supplementation with Thr in a high-fat diet [9]. The disruption of lipid metabolism can contribute to cardiovascular disease. Threonine may be a positive modulator of lipid metabolic disorders. One study discovered that plasma Thr level is linked to a lower risk of having an atherogenic lipid profile in humans, an obvious negative correlation between Thr and levels of small dense low-density lipoprotein cholesterol (sdLDL-C) and triglycerides (TG) [29].

However, emerging evidence suggests that moderate dietary protein restriction is beneficial for metabolism in animals and humans [30,31]. Many studies have indicated that restriction of essential amino acids and non-essential amino acids can promote lipid metabolism and resist obesity through various pathways [32,33]. One study found that dietary Thr restriction helps to prevent the metabolic disorders associated with obesity [34]. Dietary Thr restriction can improve metabolism by regulating hormone liver fibroblast growth factor 21 (FGF21) [34]. FGF21 increases lipolysis in white adipose tissue and substrate consumption in the liver, making it an effective modulator of fatty acid oxidation and lipid metabolism [35].

### 3.2. Protein Synthesis

Threonine is required for the synthesis of Thr-rich proteins in epithelial tissues, such as mucins [36]. Mucin synthesis is quite sensitive to dietary Thr concentration, where a deficiency of or excessive dietary Thr reduces the production of gut mucosal protein and mucins in animals [37]. Apart from intestinal mucin, Thr also has beneficial effects on protein synthesis in other tissues such as skeletal muscles. Studies on different aquatic animals and livestock have reported that Thr can promote growth and enhance the protein synthesis of skeletal muscle [37,38]. Threonine not only serves as a precursor for protein synthesis but also as a signaling molecule that can regulate the protein synthesis pathway. Insulin-like growth factor I (IGF-I), an upstream activator of mammalian target of rapamycin (mTOR) in skeletal muscle, is essential for muscle development and regeneration [39]. The mTOR is a downstream component of the PI3K/AKT pathway, which modulates protein synthesis through ribosomal S6 kinase (S6K) and the eukaryotic translation initiation factor 4E-binding protein 1 (4E-BP1) [40]. A recent publication reported that dietary Thr promoted muscle protein synthesis through activating the PI3K/AKT/TOR signaling cascade via IGF-I in fish [41]. As a precursor of glycine, Thr is degraded by TDH to generate glycine. Glycine modulates protein synthesis by activating mTORC1 in a PI3K/Akt-dependent manner and suppressing proteolysis [42].

## 4. The Effects of Thr Metabolism on ESC Function

### 4.1. Proliferation and Differentiation

Embryonic stem cells are pluripotent stem cells that can be driven to differentiate into almost all cell types [43]. With adequate nutritional support, ESCs can self-renew indefinitely while maintaining pluripotency in culture. One of the main approaches to fully exploit the therapeutic potential of ESCs is to develop strategies for differentiating them into particular lineages [44]. It is apparent that the medium’s nutritional constituents are responsible for differentiation, and nutrient content may be used to specify lineages [44]. Many studies reported that amino acids including Thr, proline, and methionine regulate ESC proliferation and differentiation in cell culture [44,45,46]. The existence of Thr in the culture media is necessary for the undifferentiated state and proliferation of mouse ESCs [47]. It is reported that the TDH mRNA levels in mouse ESCs were 1000 times greater than in differentiated cells [10]. Threonine generates glycine and acetyl-CoA under the action of TDH, which is required for mouse ESC viability [43]. Inhibition of TDH activity induces the selective death of mouse ESCs through autophagy [47]. Glycine can help promote the purine and pyrimidine (thymidine) production required for mouse ESC proliferation by contributing to one-carbon metabolism, while acetyl-coA enters the TCA to eventually produce ATP [48]. Supplementing extra glycine and pyruvate in the culture media can reverse the death of mouse ESCs caused by Thr depletion, by maintaining the one-carbon metabolism and providing acetyl-CoA [49]. Therefore, the TDH-mediated Thr metabolic pathway provides the substrates needed for rapid ESC proliferation. Furthermore, TDH-mediated Thr catabolism is involved in nutrient utilization ways that support chromosomal DNA replication and the cell cycle process in mouse ESCs [44]. When ESCs are exposed to Thr-deficient medium or TDH inhibitors, nucleic acid synthesis stops immediately and the cell cycle phase is blocked at the G1 phase [50]. Threonine activates signaling pathways including PI3K/Akt, MAPKs, mTOR, p7056k, and 4E-BP1 to stimulate ESC transition through the G1/S phase [50]. Moreover, Thr also has an effect on the ESC differentiation. Knockdown of TDH activity by small interfering RNA results in the increased expression of markers reflecting differentiation [49].

### 4.2. Epigenetic Regulation

Epigenetic regulation is considered to be a key step in ESC differentiation. TDH-mediated Thr catabolism participates in epigenetic regulation (methylation and acetylation) for mouse ESC self-renewal [49]. Mouse ESCs need to activate chromatin modifications such as the trimethylation of histone H3 lysine 4 (H3K4me3) to regulate and maintain proliferation and a pluripotent state [46,51]. S-adenosylmethionine (SAM), which is the methyl donor for most biosynthetic processes and is produced by the one-carbon metabolism, is responsible for histone methylation [14]. The suppression of Thr catabolism by Thr deprivation or TDH knockdown in ESCs can reduce SAM concentration, and, consequently, selectively suppress Di- and trimethylation of H3K4 [49].

## 5. The Roles of Thr on Intestinal Health and Functions

### 5.1. Intestinal Thr Uptake and Utilization

The studies based on isotopic tracer techniques have revealed that from 20% to 70% of dietary essential amino acids are absorbed by the portal-drained viscera (PDV) in the first-pass metabolism of pigs [19]. There are approximately 40–60% of Thr from the diet were intercepted by the intestine during the first pass [15,52]. Bertolo et al. [52] identified that the whole-body Thr need was reduced by 60% in piglets receiving total parenteral nutrition (TPN) compared to piglets receiving enteral nutrition, which suggested that Thr exerted an indispensable function in the gut. Dietary Thr deficiency leads to a decrease in the quantity of intestinal goblet cell and mucin content, which cannot be restored by intravenous Thr; thus, this implies that the gut utilizes substantial amounts of Thr sourced from the intestinal lumen rather than arterial blood [53]. The absorption of nutrients is mediated by specific transporters and digestive enzymes (alkaline phosphatase, Na+/K+- atp enzyme, aminopeptidase N, sucrase isomaltase) that exist on the brush membrane of the intestinal epithelium [54,55]. The aminopeptidase N digests peptides by N-terminal cleavage, especially those formed from neutral and basic amino acids including Thr [56]. Dietary Thr was found to enhance Na+/K+-ATPase activity [38]. Transporters regulate amino acid concentrations by regulating extracellular and intracellular amino acid transition [57]. The uptake and transport of luminal Thr is regulated by amino acid transporters in intestinal epithelial cells. B^0^AT, ASCT1, and y^+^LAT1 are linked to Thr transport. Dietary Thr is considered to be rapidly absorbed by B^0^AT transporters at the brush border membrane of the small intestine [58]. The y^+^LAT1, a Na+-dependent transporter located at the basolateral membrane, exchanges neutral amino acids (including Thr) for intracellular cationic amino acids [54]. ASCT1 transports small neutral amino acids such as Thr, serine, cysteine, and alanine [59]. The level of Thr can affect the expression of the transporters. One study found that Thr deficiency up-regulated ASCT1 expression in broilers infected with coccidiosis, which suggests that adequate Thr is required in the intestine [60].

The dietary Thr absorbed by the gut is used by the body for protein synthesis or oxidation for energy production. The gut is the key site for amino acid utilization and metabolism [55,61]. Threonine not only takes part in the synthesis of mucosal proteins, but is also catabolized by luminal bacteria in the intestine [15]. Threonine used for oxidation in the gut of piglets only accounts for 2–9% of the total Thr, but its use for the synthesis of mucosal proteins accounts for 71% of total Thr usage [19]. The peptide backbone of mucin is notably high in Thr, which accounts for 30% of the total amino acids in this protein. As dietary Thr becomes limited, muscle growth and other tissue functions are restricted at the expense of maintaining mucin production [62]. Certain commensal bacteria (*Clostridium* species) in the gut may degrade Thr, resulting in the formation of volatile fatty acids (acetic acid, propionic acid, and butyric acid) that are required for maintaining intestinal function and modulating immunological responses [63,64].

### 5.2. The Effects of Thr on Intestinal Health and Function

#### 5.2.1. Nutrient Digestibility

The gut has various roles, including nutrient digestion and absorption as well as immune defense against pathogens and toxins [65,66]. The integrity of the intestinal structure is the foundation for the intestinal function of nutrient uptake and absorption. Adequate Thr is taken and utilized by the intestinal mucosa, and contributes to the maintainance of mucosa integrity. In the small intestine of broilers, dietary Thr supplementation has a positive effect on villus height, crypt depth, epithelial thickness, and the quantity of goblet cells [67,68]. Deficient or excessive dietary Thr reduces villous area, induces villous atrophy, and increases the apoptosis rate of intestinal epithelial cells in weaned piglets [6,69]. The higher villus contributes to providing more surface area for nutrient intake [68]. In addition, the Thr demand for amylase synthesis is particularly high, accounting for around 11% of total protein. Studies have discovered that a lack of Thr in the diet reduces the synthesis of digestive enzymes and the activity of brush border enzymes in the animal gut [38,70]. It is therefore possible that Thr may aid in enhancing intestinal digestive and absorptive capacity.

#### 5.2.2. Gut Microbiota

Dietary Thr has a positive impact on gut microbiota. High dietary Thr (26% higher than the NRC recommendation, 1994) reduced *Salmonella* and *Escherichia coli* (*E. coli*) colonies, while increasing *Lactobacillus* in broiler chickens [2]. In addition, studies have shown that when under stress, dietary Thr reequilibrates the gut microbiota of animals. A low crude protein diet supplemented with Thr (at 0.3%) recovered the microbial diversity and significantly enhanced the abundance of potentially beneficial microorganisms in laying hens [71]. An amino acid cocktail containing L-Thr increased the frequency of beneficial populations of *Enterobacteria*, *Lactobacillus*, *Bacteroides*, and *Enterococci* in rats challenged with dextran sodium sulfate [72]. One of the explanations for this effect might be that Thr promotes mucin secretion, because mucins cannot be digested in the small intestine and, therefore, arrive to the large intestine, where they serve as the substrates for saccharolytic bacteria [73]. Koo et al. [74] found that the supplementation of Thr 15% higher than the NRC recommendation can interact with feed composition to alter gut microbial fermentation. The data also imply that Thr has beneficial effects on intestinal microbiota which might be related to mucin synthesis and immunoglobulin production as a result of Thr addition [72].

#### 5.2.3. Barrier Function

The mucus layer that covers the intestinal epithelium is a vital aspect of the intestinal barrier function [75]. The mucus layer is a physical layer that contains mucins, immunoglobulins, salts, antimicrobial substance, etc., which is secreted by different glands. The mucus layer serves as the frontline for preventing damage caused by digestive enzymes, microbes, and pathogens [76]. Mucin-2 (MUC2) is the major constituent of intestinal mucus, which is produced primarily by goblet cells. MUC2 is a highly glycosylated glycoprotein with a central protein backbone rich in Thr and serine, and is connected to multiple O-linked oligosaccharide side chains, leading to high resistance to proteolysis [77]. Threonine is the main factor to regulate intestinal mucin secretion. Compared with adequate Thr (0.89%), both a lack and excess of (0.37% and 1.11%) Thr significantly affects the amount and type of intestinal mucin in piglets [6]. However, it is not clear how excessive dietary Thr reduces the synthesis of intestinal mucosal protein and mucins. One possible explanation is that neutral amino acids (including branched-chain amino acids) share the same transport systems with Thr, which restricts its intestinal intake, thus limiting the protein synthesis [37,78]. This possible explanation requires further study. Increasing evidence has shown that goblet cell differentiation and mucin production are sensitive to dietary Thr concentration [36,53]. When dietary Thr supplementation was higher than growth requirements, goblet cell density and MUC2 mRNA expression were enhanced in gut [2,79]. One study indicated that dietary Thr could affect goblet cell differentiation through modulating the expression of genes associated in the Notch-Hes1-Math1 pathway, which promoted the MUC2 synthesis [80]. The Notch signaling pathway is essential for intestinal cell differentiation [81]. Notch signaling activates the Hes1 transcription factor and inhibits the bHLH transcription factor Math1 (the human homologue is Hath1) [82]. Hath1 activation causes MUC2 induction by binding to its binding sites, Eboxes on the MUC2 promoter [83,84]. MUC2 has the dual functions of a physical barrier and immune regulation, and can interact with epithelium, microbiota, and the host immune system to maintain intestinal homeostasis. Moreover, the effect of Thr on mucin synthesis is related to the age and physiological state. It was found that 45% of the dietary Thr requirement was needed to maintain intestinal mucosa in 3-day-old pigs [52]. Research on animals has shown that under conditions of disease and stress, such as ileitis, sepsis, inflammation, and intrauterine growth retardation (Table 1), the Thr requirement in the intestine is greatly raised in order to promote mucin production [80]. This suggests that intestinal inflammation increases the production of mucus to protect the gut. In conclusion, dietary Thr supplementation stimulates the synthesis of MUC2, making MUC2 exert a stronger effect on the gut barrier and immune function, and maintain intestinal homeostasis.

In addition, many recent studies have focused on the influence of the relationship between dietary Thr and dietary fiber on intestinal barrier function [85,86,87]. Dietary fiber has positive effects on barrier function by boosting mucus secretion and mucin synthesis [88]. However, high dietary fiber increases the loss of endogenous amino acids, resulting in a greater need for Thr to maintain mucin production [85,89]. The effect of Thr on intestinal barrier protein is influenced by the amount of fiber in the diet. It was found that the expression of barrier protein rose as Thr increased in pigs fed a low-fiber diet, not a high-fiber diet [90].

#### 5.2.4. Immune Function

**Figure 2:**
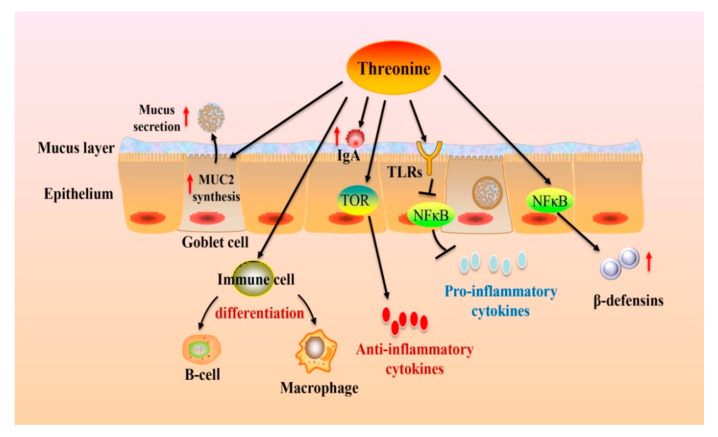
Dietary Thr modulates the intestinal immune function. Dietary Thr affects goblet cell differentiation, which, in turn, stimulates MUC2 synthesis. Thr regulates immune cell differentiation and immunoglobulins (Ig) production. Meanwhile, Thr can modulate the release of cytokines via the TOR and NF-κB pathways. Abbreviations: IgA: immunoglobulins A; MUC2: mucin-2; TLRs: toll-like receptors; TOR: the target of rapamycin; NF-κB: nuclear factor κ-light-chain-enhancer of activated B cells.

Numerous reports have shown that dietary Thr is essential for maintaining gut immune function (Figure 2) [93,94]. The immune function depends on its innate immunity and adaptive immunity response in the gut. The adaptive immunity depends on lymphocytes proliferation and immunoglobulins (Ig) production. In vitro, Thr was proven to be used by lymphocytes to support their proliferation and antibody secretion [60]. K203 is a CC chemokine that attracts macrophages and monocytes [95]. C-C chemokine receptor type 9 (CCR9) is found mostly on gut-homing T lymphocytes and induces the formation of gut lymphoid tissue [96]. C-X-C chemokine receptor type 5 (CXCR5) is expressed on B cells and B-helper T cells and is necessary for intestinal immunity [97]. Threonine deficiency leads to enhanced K203 and CXCR5 mRNA expression, and reduced CCR9 mRNA expression, showing that immune cells tend to transfer to B cells and macrophages instead of T cells [60]. Moreover, Thr is also a vital element of Ig, taking up to 7–11% of total amino acids, especially IgA. The polymeric immunoglobulin receptor (pIgR) is present on the basolateral surface of intestinal epithelium and is responsible for transporting IgA to the lumen through epithelial cells [98]. Threonine can regulate pIgR concentration by NF-κB activation to influence IgA production under stress [60,93].

Thr not only regulates lymphocytes and IgA secretion but also modulates the expression of inflammatory cytokines. In the animal intestine, Thr supplementation up-regulates inflammatory factors interleukin *(IL)-6* gene expression in piglets [80], down-regulates the expression of inflammatory mediators interferon γ (*IFN-γ*) and *IL-12* in the broilers [2,99], and tumor necrosis factor alpha (*TNF-α*) in fish [16]. The levels of inflammatory factors, such as *IL-8*, *IL-6*, *IL-1β*, inducible nitric oxide synthase (*iNOS*), *TNF-α*, and *IFN-γ* are significantly decreased in stress-related mucosal disease of rats pretreated with Thr [100]. When fish are infected with bacteria, Thr deficiency will aggravate an intestinal inflammatory response by promoting pro-inflammatory cytokine *TNF-α*, *IL-1β*, *IFN-γ2*, *IL-6*, *IL-8*, and *IL-1*7 gene expression through the NF-κB signaling cascade, and inhibiting the expression of anti-inflammatory cytokines *TGF-β1, TGF-β2, IL-4/13A,* and *IL-10* via the TOR complex signal pathway [16,101]. Futhermore, Thr supplementation may suppress the inflammatory cytokine synthesis by inhibiting the TLR4 signal pathway to improve intestinal immune function [7]. Antimicrobial peptides in the gut also contribute to the intestinal immune response [102]. A recent study found that L-Thr at the concentration of 1mM upregulates β-defensin (pBD-1, pBD-2, and pBD-3) expression by activating NF-κB signaling and inhibiting sirtuin-1 (SIRT1) expression in porcine intestinal epithelial cells [103]. In addition, the interaction between Thr and fiber can enhance the immune function. Research showed that Thr and pectin had a synergistic action on intestinal immune response in broilers infected with coccidiosis [99]. Moreover, dietary Thr supplementation with the addition of sugar beet pulp drove the antibody titer to a high level [86].

## 6. Conclusions

Threonine is an indispensable amino acid involved in lipid metabolism, protein synthesis, ESC proliferation and differentiation, and intestinal health and function. The requirement for and metabolism of Thr are closely related to health and disease in animals. Appropriate Thr contributes to relieving energy metabolism disorder and intestinal inflammation. However, Thr effects involve the regulation of nutrition metabolism, and further studies are needed to validate the findings in different animal models. Moreover, further experiments are required to determine how Thr regulates the dynamic balance between intestinal microbiota, immune response, and barrier.

## Figures and Tables

**Table 1:** The effects of Thr levels on intestinal barrier function in animals under pathological conditions.

Treatment	Study Design	Main Findings	Reference
0.57%, 1.07%, and 2.07% dietary Thr	male Sprague-Dawley rats, 10 months, *n* = 8/treatment, 20 days, intestinal inflammation	Increased the number of goblet cells and enhanced mucin synthesis and the mucosal mass	[36]
140 mg/kg/d Thr, intragastric administration 4 µmol/kg/d L-[15N]Thr, intravenous administration 4 µmol/kg/d L-[U-13C]Thr	Pitmann-Moore minipigs, 10 months, *n* = 4/treatment, 7 days, ileitis	Promoted intestinal mucin synthesis and PDV utilization of Thr	[91]
intravenous administration 500 µmol/100 g weight L-[ U-13C]Thr	male Sprague-Dawley rats, 300 g body weight, *n* = 12/treatment or 14/treatment, 6 days, sepsis	Promoted the synthesis of mucin and mucosal protein	[92]
the basal diet supplemented with 3 · 0 g/kg L-Thr	male Arbor Acres Plus broilers, 1 day, *n* = 48/treatment, 21 days, lipopolysaccharide -challenged	Enhanced intestinal goblet cell density and MUC2 mRNA expression	[7]
the basal diet supplemented with 2.0 g/kg L-Thr	piglets, newborn, *n* = 18/treatment, 21 days, piglets with intrauterine growth retardation	Increased the production of MUC2 and the density of goblet cells	[80]

MUC2, mucin-2; PDV, portal-drained viscera.
